# EGFR Inhibitor Erlotinib Delays Disease Progression but Does Not Extend Survival in the SOD1 Mouse Model of ALS

**DOI:** 10.1371/journal.pone.0062342

**Published:** 2013-04-26

**Authors:** Claire E. Le Pichon, Sara L. Dominguez, Hilda Solanoy, Hai Ngu, Nicholas Lewin-Koh, Mark Chen, Jeffrey Eastham-Anderson, Ryan Watts, Kimberly Scearce-Levie

**Affiliations:** 1 Department of Neuroscience, Genentech Inc., South San Francisco, California, United States of America; 2 Department of Pathology, Genentech Inc., South San Francisco, California, United States of America; 3 Department of Nonclinical Biostatistics, Genentech Inc., South San Francisco, California, United States of America; University of IIllinois, United States of America

## Abstract

Amyotrophic lateral sclerosis (ALS) is a fatal neurodegenerative disease that causes progressive paralysis due to motor neuron death. Several lines of published evidence suggested that inhibition of epidermal growth factor receptor (EGFR) signaling might protect neurons from degeneration. To test this hypothesis in vivo, we treated the SOD1 transgenic mouse model of ALS with erlotinib, an EGFR inhibitor clinically approved for oncology indications. Although erlotinib failed to extend ALS mouse survival it did provide a modest but significant delay in the onset of multiple behavioral measures of disease progression. However, given the lack of protection of motor neuron synapses and the lack of survival extension, the small benefits observed after erlotinib treatment appear purely symptomatic, with no modification of disease course.

## Introduction

Amyotrophic lateral sclerosis (ALS) is a devastating neurodegenerative disease primarily affecting motor neurons. It causes rapid progressive paralysis, with 80% mortality 2–5 years following diagnosis. Worldwide, the incidence of ALS is 1 in every 50,000 people per year (www.alscenter.org). Riluzole, the current standard of care for ALS, only extends lifespan by 2–3 months and has undesirable side effects such as nausea and fatigue [1]. Developing a successful drug for ALS represents an urgent and significant unmet medical need.

The SOD1^G93A^ mouse model of ALS is the most widely used animal model for ALS as it phenocopies many aspects of the human disease [2]. In these mice, a familial mutation in the human SOD1 gene (G93A) that causes ALS is expressed transgenically throughout the body under the control of the endogenous mouse SOD1 promoter. The transgene insertion causes a degenerative disease of lower motor neurons leading to progressive paralysis and eventual death, with the number of transgene copies correlating with severity of disease [3].

In these mice the earliest documented pathological event is denervation of motor neurons from fast-twitch muscle fibers [4], followed by degeneration of motor nerves and motor neuron cell body death [2], and ultimately the loss of connected interneurons [5]. This neuronal pathology is accompanied by inflammation in the peripheral nerves, spinal cord and brainstem [6], [7], [8], [9]. At the behavioral level, early symptoms include loss of full hind limb extension, loss of grip strength, and appearance of tremor and gait abnormalities [2], [10], [11], [12], [13]. These symptoms eventually progress to complete paralysis and early death.

Several lines of evidence suggested that the epidermal growth factor receptor (EGFR) signaling pathway could play a role in the pathology of neurodegenerative conditions in general and specifically in ALS. Treatment with EGFR inhibitors is reportedly neuroprotective in both a rat model of glaucoma [14] and a rat model of spinal cord injury [15]. In both studies the authors suggest that EGFR inhibition targets reactive astrocytes. Furthermore, EGFR mRNA expression was found to be upregulated over 10-fold in the spinal cord of human ALS patients as well as in that of the SOD1^G93A^ mouse model [16], suggesting that pharmacological inhibition of EGFR signaling could be a feasible strategy to slow progression of this disease.

Erlotinib, an EGFR inhibitor marketed for the treatment of non-small cell lung carcinoma, presented an opportunity to determine if inhibition of this pathway would also have a beneficial effect in the SOD1^G93A^ mouse model of ALS. To our knowledge, this type of treatment has not previously been tested in this mouse model.

In our study, erlotinib penetrated into the central nervous system and resulted in a modest yet statistically significant symptom delay as measured by multiple readouts of disease onset and progression. However, this treatment failed to extend lifespan, did not protect motor synapses, and did not correlate with a modulation of markers for astrocytes and microglia. We thus conclude that erlotinib is not efficacious in treating the SOD1 mouse model of ALS.

## Materials and Methods

### Study Design

To examine the effect of erlotinib treatment in the SOD1 mouse model, we designed two complementary studies. In a survival study we examined behavior and lifespan (n≥46 per treatment group; Table 1), and in a histology study we examined motor neuron synapses at an early stage of disease (n = 12 per treatment group; Table 1).

**Table 1:** Animal n per treatment group in each study.

	SOD1 genotype	Vehicle n	Erlotinib n	Total n
**Survival study**	**Tg**	46	48	94
	**WT**	6	6	12
	**Combined**	52	54	106
**Histology study**	**Tg**	12	12	24
	**WT**	6	4	10
	**Combined**	18	16	34

**Figure 1:**
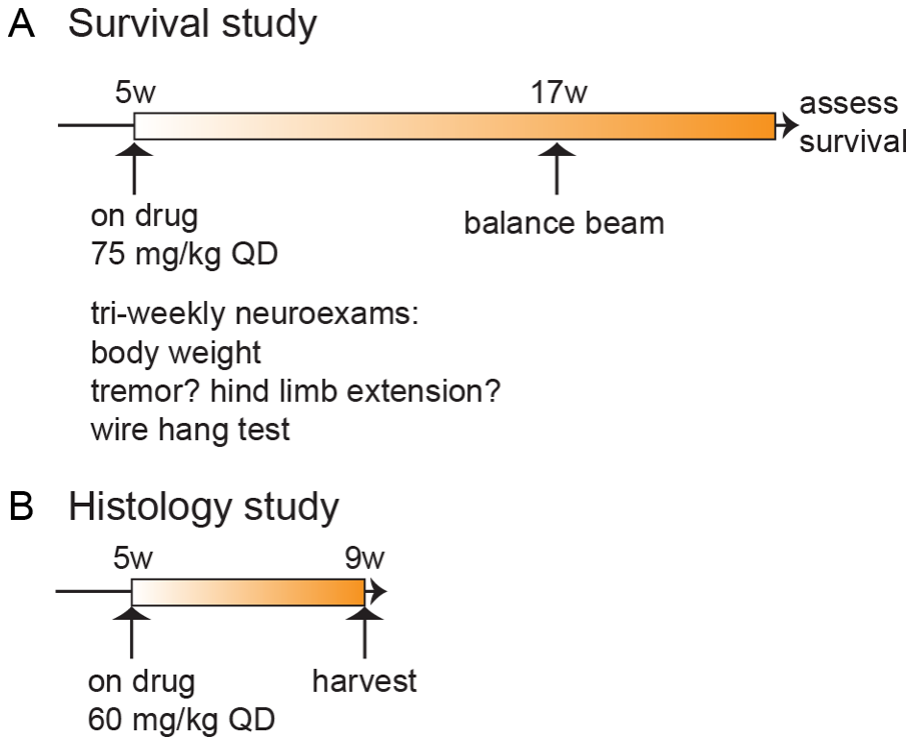
Study design and timelines. (A) Study 1: timeline for survival study. n = 106 mice (see Table 1 for breakdown of n per genotype and treatment) received daily IP doses of erlotinib from 5 weeks of age onwards, and their lifespan was measured. Mice were assessed in neurological exams 2 times/week between 6–9 weeks, 3 times/week from 9 weeks of age onwards, and tested on the balance beam at 17 weeks of age. (B) Study 2: timeline for histological endpoints study. Mice (n = 34; see Table 1 for breakdown by genotype and treatment) were dosed from 5–9 weeks of age, and euthanized for tissue collection at 9 weeks.

In the survival study we treated SOD1 mice daily with 75 mg/kg erlotinib or vehicle IP (intraperitoneally) from 5 weeks of age until they reached criteria for euthanasia (Figure 1A). The mice tolerated this daily IP regimen over 4+ months. The survival study design incorporated best practices recommended in Scott et al., 2008 [17]. In the histology study we treated SOD1 mice daily with 60 mg/kg erlotinib IP during a 4-week window (between 5 and 9 weeks of age; Figure 1B) and harvested tissue from the animals at the end of the dosing window. For both studies, although twice-daily dosing would have better maintained systemic erlotinib levels, we decided not to subject the mice to such a large number of injections, especially considering the length of the studies.

### Mice

SOD1^G93A^ high copy number transgenic mice (SOD1 Tg) and their non-transgenic littermates (SOD1 WT) were bred at Genentech. They were originally derived from Jackson Laboratory (Bar Harbor, ME; stock #002726, [2]) and backcrossed for at least 10 generations into C57BL/6N (Charles River).

Mice were housed on a regular light/dark cycle (14∶10 hours) with ad libitum access to food (LabDiet 5010) and water. Up to 5 mice were housed in each 7″×11″×5″ Micro-VENT cage (Allentown) with 21 air changes/hour, and Enrich-O’Cobs bedding (The Andersons, Maumee OH). When mice started taking longer than 1 s to right themselves when placed on their side, soft food (DietGel® 76A, PharmaServ) was placed on the cage floor as well as a gel pack (HydroGel®, PharmaServ) for hydration. Any cage mates of these mice also had access to this food and water on the bedding surface.

The treatment groups were balanced for sex and litter and were staggered into 4 cohorts by birth date across 2.5 months. After balancing, assignment to treatment was random. For dosing convenience, the animals were caged by treatment.

The protocols for mouse experiments were approved by the Genentech Animal Care and Use Committee. All work was conducted according to NIH guidelines for the humane care and treatment of laboratory animals.

### Drug Formulation

Erlotinib was freshly formulated every 3–4 weeks as needed by suspending erlotinib powder at 10 mg/ml in a vehicle solution of 30% Captisol® (a modified β-cyclodextrin facilitating drug solubilization). The weight of erlotinib powder needed was calculated according to the following formula: 10 (mg/mL)/0.915×V (mL) = W (mg), where V is the volume of formulation prepared, W is the weight of erlotinib powder needed, and 0.915 is the salt correction to attain free base equivalency. The mixture was sonicated for 40 minutes with occasional vortexing until a clear solution was obtained. The pH was adjusted from roughly pH 4.0 to pH 4.5 by adding NaOH (if the pH is adjusted higher than 5.0 the compound may precipitate). The formulation was filtered through a 0.45 µm cellulose acetate filter to remove any visible particulate matter into an amber glass vial and stored at 2–8°C.

### Dosing

Mice were dosed IP daily at 60 or 75 mg/kg (histology study and survival study respectively) at least 1 hour prior to any behavioral testing. The exact injection site and side were varied from day to day so as to minimize tissue scarring related to repeated needle entry to the same site. To streamline the dosing process, mice were assigned to a weight bracket with each bi-weekly weighing, which corresponded to a given dosing volume. This was equivalent to rounding the exact dosing volume to the nearest 20 µl (80, 100, 120 µl and so on). The actual dose ranges were thus 60 mg/kg +/−7 or 75 mg/kg +/−5 due to weight variations.

Injections were discontinued once mice reached within 1 g of a 20% weight loss from their peak weight in order to minimize handling stress and discomfort for these end-stage animals.

### Blood Sampling and Serum Separation

To measure peripheral exposure of erlotinib in the mice, up to 100 µl blood was sampled from mice via the retro-orbital plexus. Mice were closely monitored following the bleed. All mice were bled regardless of treatment. The blood was collected in BD Microtainer® tubes (Cat# 365956, BD Microtainer) and allowed to clot at room temperature for >30 minutes before spinning for 10 minutes at 8,000 g. The serum was pipetted into clean tubes and frozen at −80°C prior to shipment to Advion BioServices Inc. (Ithaca, NY) for analysis of erlotinib levels.

### Spinal Cord Harvest for Analysis of Erlotinib Content

Mice were euthanized and a cut was made at either end of the spinal column. Spinal cords were harvested by inserting an 18cc needle into the caudal end of the column and flushing the tissue out of the cervical end with a PBS-filled syringe. Samples were immediately frozen and stored at −80°C.

### Measurement of Erlotinib Levels in Mouse Serum and Spinal Cord

Erlotinib content in mouse serum samples were determined by LC-MS (liquid chromatography-mass spectrometry). Separation of erlotinib was achieved using a Waters Symmetry C18 reversed-phase analytical column (3.5 µm particles, 4.6×50 mm, #WAT200625). Analytes were then run on a mass spectrometer (Sciex API 3000) for identification. The method was similar for the spinal cord sample analysis except that they were run on a Phenomenex Kinetex C18 column (2.6 µm particles, 2.1×50 mm, #00B-4462-AN). The mass spectrometer was AB Sciex 5500Qtrap. The lower limit of quantitation for both assays was 1 ng/ml.

### Criteria for Euthanasia

Once an animal had lost 15% peak body weight, it was checked daily, and gel food and gel water were provided on the bedding. Euthanasia criteria were the inability of the mouse to right itself within 15 seconds or a 20% decrease from peak body weight.

### Tissue Harvest and Preparation

#### SOD1 mice

Mice were anesthetized with 2.5% tribromoethanol (0.5 ml/25 g body weight) and transcardially perfused with phosphate-buffered saline (PBS) for exsanguination followed by 4% paraformaldehyde (PFA) in PBS for fixation. Gastrocnemius and soleus muscles were collected from both legs of each mouse and were cryoprotected in 30% sucrose. Spinal cords were harvested as previously described [18] and post-fixed in 4% PFA overnight, then transferred to PBS and shipped to NeuroScience Associates (Knoxville, TN) for histology.

#### EGFR KO and WT embryos

E16.5 embryos were harvested and perfused with PBS then drop-fixed in 4% PFA overnight, followed by cryoprotection in 30% sucrose. 20 µm-thick cryosections of whole heads were made. Genotypes were confirmed using a tail clip.

### Spinal Cord Histology

#### Spinal cord sectioning and sampling

Lumbar spinal cord segments were treated overnight with 20% glycerol and 2% dimethylsulfoxide to prevent freeze artifacts and multiply embedded into a gelatin matrix using MultiBrain® Technology (NeuroScience Associates, Knoxville, TN). After curing, each block was rapidly frozen by immersion in isopentane chilled to −70°C with crushed dry ice and mounted on a freezing stage of an AO 860 sliding microtome. Each MultiBrain® block was sectioned coronally at 25 µm. All sections were collected sequentially into a series of 24 containers filled with Antigen Preserve solution (50% PBS pH 7.0, 50% ethylene glycol, 1% polyvinyl pyrrolidone).

The cords were sectioned coronally at 25 µm thickness. A series of 33 sections equally spaced at 300 µm intervals throughout the entire lumbar spinal cord was used for staining.

#### Spinal cord immunohistochemistry

The sections were stained free-floating. All incubation solutions from the blocking serum onward used Tris buffered saline (TBS) with Triton X-100 (TX) as the vehicle; all rinses were with TBS.

After a hydrogen peroxide treatment and blocking serum, the sections were immunostained overnight at room temperature with Goat anti-ChAT (choline acetyl-transferase; Millipore AB144P) at 1∶1000, Rabbit anti-GFAP (glial fibrillary acidic protein; Dako Z0334) at 1∶20,000, Rabbit anti-Iba1 (ionized calcium binding adaptor molecule 1; Wako 019–19741) at 1∶15,000, or Rabbit anti-phospho-EGFR (pEGFR; Novus NB110–56945) at 1∶100,000. Vehicle solution contained TritonX-100 for permeabilization. Following rinses, a biotinylated secondary antibody (Vecta anti-goat or anti-rabbit elite) was applied at 1∶250. To visualize the location of binding site of the primary antibody, an avidin-biotin-HRP complex (details in Vectastain elite ABC kit, Vector, Burlingame, CA) was applied. After rinses, the sections were treated with diaminobenzidine tetrahydrochloride (DAB) and hydrogen peroxide to create a visible reaction product and mounted on gelatinized (subbed) glass slides, air dried, dehydrated in alcohols, cleared in xylene and coverslipped.

#### Motor neuron counts

Imaging of mouse spinal cord tissues on glass slides was performed at 100X magnification using Surveyor software (Objective Imaging, Cambridge, UK) controlling a DM6000B upright microscope (Leica Microsystems, Wetzlar, Germany) equipped with a robotic slide loader (Prior Scientific, Rockland, MA). Images were analyzed using Matlab with the image processing toolbox (Mathworks, Natick, MA). Binary masks of tissue sections were created using RGB color detection and morphological operations. Motor neuron cell bodies were detected automatically by applying the following steps: a) global color thresholds and morphological opening, b) close-open with reconstruction, c) regional minimal detection and watershed segmentation followed by dilation and size filtering. All image analysis was performed blind to treatment condition.

#### Positive pixel area analysis for quantitation of IHC stains

Spinal cord sections on GFAP, Iba1 and phospho-EGFR stained slides were imaged and detected as described above. Tissue masks were slightly eroded to avoid edge effects. Positive pixels were segmented using color thresholds and a brown detection algorithm [19]. Neighboring binary pixels were filtered and merged via morphological opening-closing. A minimum size cutoff of 180 squared microns was applied in the analysis of Iba1 clusters and enlarged cells. The percent area stained was calculated by normalizing the total positive pixel to tissue area. All image analysis was performed blind to treatment condition.

### Muscle Histology

#### Muscle sectioning and sampling

Each muscle was longitudinally cryosectioned at 20 µm thickness and every other section was collected. Two series of 5–6 sections, equally spaced at 480 µm intervals across the entire muscle, were used for staining and scoring.

#### Neuromuscular junction immunofluorescent staining

All steps were at room temperature unless indicated otherwise. 20 µm-thick muscle cryosections were hydrated in PBS (2×10 min) followed by permeabilization in 0.1% TritonX in PBS (PBSTx; 2×10 min). Sections were blocked for 1–2 hours in 5% normal goat serum (NGS; Vector #S-1000) in PBSTx and incubated for 48 hours at 4°C in guinea pig anti-VAChT (vesicular acetylcholine transporter; Millipore #AB1588) diluted 1∶100 in 0.5% NGS in PBSTx. Slides were washed in PBSTx (4×10 min) then incubated in Alexa488-conjugated α-bungarotoxin (Life Technologies B13422) and AlexaFluor® 594 Goat anti-Guinea pig (Life Technologies A11076), both diluted at 1∶1000 in PBSTx. Slides were washed in PBSTx (2×10 min) then in PBS (2×10 min). Slides were coverslipped with ProLongGold with DAPI (Life Technologies #P36934). Slides were allowed to set overnight at room temperature in the dark before longer-term storage at 4°C.

#### Neuromuscular junction analysis

NMJ innervation was scored manually and blind to treatment. For each NMJ identified by positive alpha-bungarotoxin staining (Alexa 488), we examined whether an apposing pre-synaptic junction labeled by VChAT (Alexa 594) could be detected. Each NMJ was scored in this manner as innervated (presence of positive VChaT staining) or denervated (absence of VChaT staining). A total of 1500–2000 NMJs from each muscle (both left and right) were scored for each animal. The average % innervation was calculated per muscle, and the two sides were averaged for each animal.

### Embryonic Brain Histology

To test the specificity of the anti-phospho-EGFR antibody (Novus NB110–56945), we stained EGFR WT and KO tissue. Adult EGFR KO adult spinal cord prepared in exactly the same manner as the SOD1 adult spinal cord would have been the ideal control, but since the EGFR KO is lethal [20] we used E16.5 embryonic brain, the oldest embryonic tissue we could harvest from matching KO and WT littermates.

All steps were at room temperature unless indicated otherwise. 20 µm-thick cryosections of whole embryonic heads were hydrated in PBS (2×10 min) followed by permeabilization in 0.1% TritonX in PBS (PBSTx; 2×10 min). Sections were blocked for 1–2 hours in 5% normal donkey serum (NDS; Jackson Immunoresearch 017-000-121) in PBSTx and incubated overnight at 4°C in rabbit anti-pEGFR Tyr1068 (Novus NB110-56945) diluted 1∶1000 in 0.5% NDS in PBSTx. Slides were washed PBSTx (4×10 min) then incubated in AlexaFluor® 488 Donkey anti-Rabbit IgG (Life Technologies A-11034) diluted at 1∶600 in PBSTx. Slides were washed in PBSTx (2×10 min) then in PBS (2×10 min). Slides were coverslipped with ProLongGold with DAPI (Life Technologies P36934). Slides were allowed to set overnight at room temperature in the dark before longer-term storage at 4°C.

### Longitudinal Behavior Measurements

All scoring was done blind to treatment.

#### Neurological exam

Body weight, body condition, grooming quality, posture and general health were assessed thrice weekly. As part of this tri-weekly assessment, both hindlimb tremor and extension reflex were assessed by suspending mice by the tail. Hindlimb symptom onset was defined as the appearance of hindlimb tremor or the inability to fully extend the hindlimbs in two consecutive observations. This early motor symptom detection is similar to the ALS Therapy Development Institute (ALS TDI) neurological score of 1 [12].

#### Wire hang

In order to quantitatively assess limb strength, mice were placed on a wire cage lid and allowed to grasp the wires. Then, the wire lid was gently inverted and held about 20 cm above the floor of the home cage. The length of time the animal was able to hang suspended was recorded up to a maximum of 60 seconds. This was repeated 3 times and the maximal score was recorded.

#### Balance beam

To assess balance and motor coordination, animals were trained to walk along an 80 cm-long wooden beam elevated 50 cm above bench surface and angled upwards. The animals traverse the beam in order to reach a dark enclosed escape box placed on a platform. Each mouse was tested on 3 different beams of decreasing diameter and increasing difficulty (large 24 mm, medium 19 mm, and small 11 mm).

Between 11–13 weeks of age, all mice were trained using the largest beam. Each mouse was first placed in the escape box for about 1 minute, then placed at the start end of the beam. Mice have a natural tendency to run upwards along the beam toward the escape box. Mice were given 8 trials over 2 days (4 consecutive trials/day) with a maximum latency of 3 minutes. If an animal paused on the beam or tried to turn and advance the wrong way, it was gently touched on the hindquarters or tail and encouraged to keep moving toward the escape box, or it was placed back at the beginning and allowed to start the trial again. Between each animal, the beam was thoroughly cleaned with soap and water followed by 70% isopropanol.

At 17 weeks of age, all mice were assessed on the balance beam in a single test session consisting of 3 consecutive trials on each of the 3 different sized beams. The latency to reach the escape box was recorded, along with the number of missteps (foot slipping off the beam). Mice were not allowed to start a trial over. If they fell off the beam, the timer was paused and they were replaced on the beam at the position where they had fallen. At least one fall on one beam occurred for n = 27 vehicle mice and n = 22 erlotinib mice. If a mouse paused for over 20 s it was encouraged to keep moving by a gentle touch on the tail or the hindquarters. The experiment was video recorded. Scoring was done live by one experimenter and also post-hoc by another experimenter using the video recording. Both experimenters were blind to treatment. As a reference for the level of difficulty of this task for a healthy age-matched littermate, examples of SOD WT data are shown in Figure S1.

### Statistical Analyses

#### Survival

To test the hypothesis that erlotinib prolongs survival in the SOD1 ALS mouse model, we fit a Weibull model to the survival curves, and tested for difference in median survival using the likelihood ratio test on the treatment parameter. While the Cox model has been suggested as the appropriate model by Scott et al. 2008 [17], we note that the survival curve for the SOD1 strain is very well characterized and in this case a parametric model has more power to discern an effect.

#### Balance beam

For the balance beam, latencies and foot slips were scored, a continuous and a count variable respectively. To analyze this data accounting for the correlation of repeated measurements on the same mouse, we fit the latencies with a linear mixed effects model and the foot slips with a generalized linear mixed effects model with Poisson error structure. The random effects in our model were animal and beam size such that we were modeling an animal-specific regression on successive beams.

#### Time to event data

For our time to event data (onset of early symptoms, weight loss, and last 60s wire hang latency) we used a combined test of the Wilcoxon and log-rank tests for censored data. We combined tests by picking the maximum test statistic of the two. We assessed significance using a permutation test on the joint distribution of the two test statistics. Our reason for using the combined test is that the log-rank test weights events equally over the entire time course, while the weighting scheme in the Wilcoxon test favors earlier events. Combining tests trades a modest loss in power for the ability to test the difference in treatments without a priori specifying how to weight observations.

#### Software

All analysis was done using R version 2.15.1 (R core team 2012) or JMP v9. For mixed models we used the lme4 package [21]. For the joint Wilcoxon-log rank test we used the surv2sample package [22]. All packages are available at CRAN (http://cran.r-project.org).

### RNA Expression Analysis

Whole spinal cords were extruded from PBS-perfused mice and each one was homogenized in 1 ml Trizol (Life Technologies, 15596-018) using a Dounce homogenizer. Total RNA was extracted following the Trizol RNA extraction protocol. cDNA was synthesized using the High Capacity RNA-to-cDNA Kit (Applied Biosystems 4387406). qPCR reactions were run on a 7500 Real Time PCR System (Applied Biosystems) using Taqman Gene Expression Master Mix (Applied Biosystems 4369016) with ∼80 ng cDNA input per reaction, EGFR Taqman assay Mm00433023-m1, and with actin B as the endogenous control gene (Mm00607939-s1). Each reaction was run in duplicate and there were n = 6 mice per genotype.

## Results

We designed two complementary studies, one to examine behavior and survival (Figure 1A) and another to examine histopathology at an early stage stage of disease (Figure 1B; further detail in Methods). In both studies, the SOD1 mice were dosed daily IP with erlotinib starting at 5 weeks of age.

### Erlotinib Fails to Extend Lifespan but Provides Modest Amelioration of Clinical Signs

**Figure 2:**
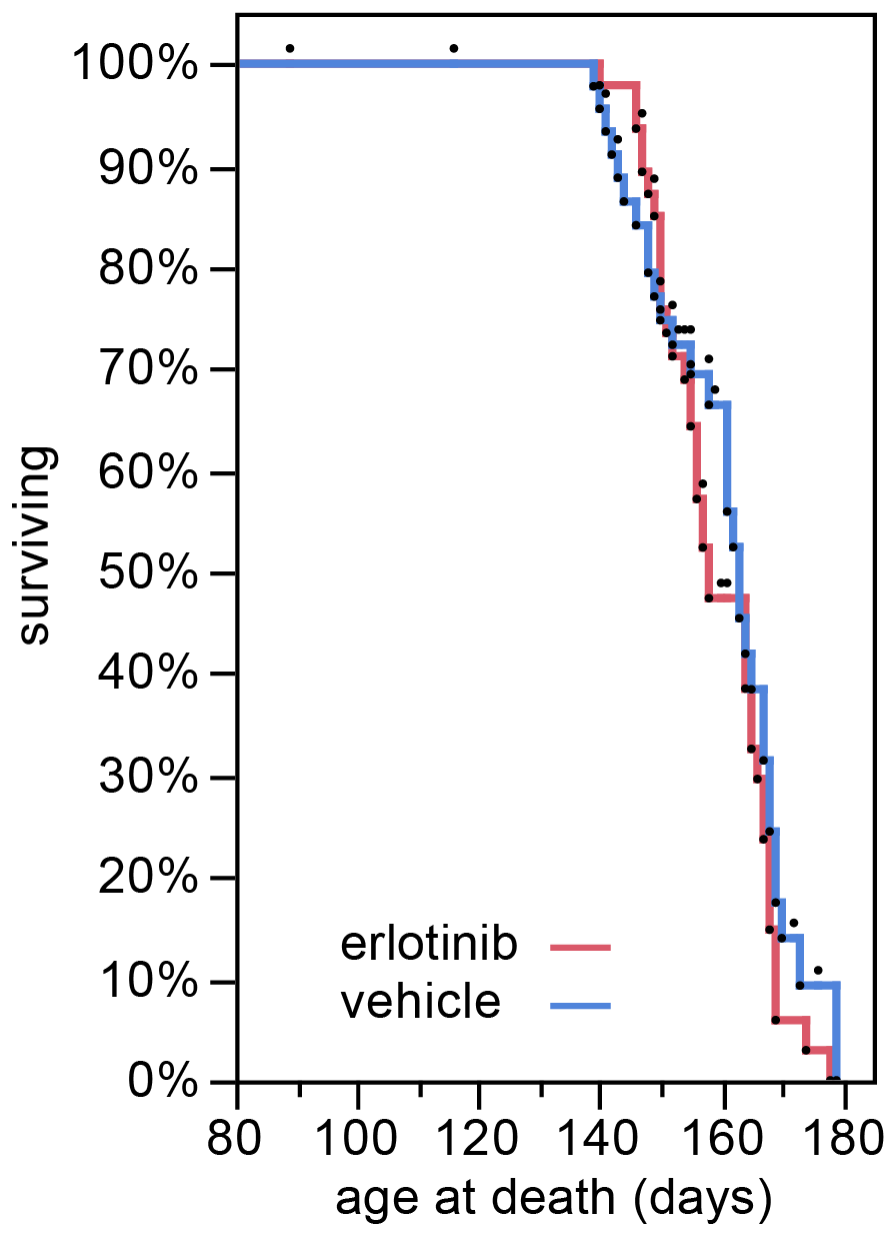
Erlotinib does not extend lifespan of SOD1 Tg mice. Kaplan-Meier survival plot showing the lifespan of the SOD1 Tg mice treated with vehicle (blue) or erlotinib (red). The lifespan of each mouse is represented by a black dot. All mice were included in the statistical analysis. Mice that were censored in the analysis because of a non-ALS death are represented by dots that are not accompanied by a lowering of the curve.

In the survival study, erlotinib treatment did not extend SOD1^G93A^ mouse lifespan, as shown by close overlap of the survival curves (Figure 2; vehicle mean survival 159.4 days, n = 46 (15 censored); erlotinib mean survival 160.8 days, n = 48 (9 censored)). Mice censored in the statistical analysis were all non-ALS deaths [17] and 22/24 were cases in which mice were found dead yet had not reached criteria for euthanasia as assessed in the previous health check (between 1–3 days prior). These late yet premature deaths were most likely related to a problematic injection. The youngest probable injection-related death was at 116 days, the mean was 153 days, and the incidence of such deaths was 24% (Table 2). Interestingly, the incidence of injection-related deaths was higher in the vehicle-treated group (n = 15 vehicle vs n = 7 erlotinib) and thus does not appear to be drug-related. Despite the relatively high rate of censoring, the groups were still balanced for litter; however, there was a slight gender imbalance in the vehicle group only, where males were more sensitive than females (12 males vs 3 females censored in the vehicle group; Table 2). The gender distribution of censored mice is listed in Table 2. Furthermore, the mean age at death of the censored mice was very close to the mean survival (153 vs 159 days), therefore the censored mice lived long enough to contribute meaningful data to the survival analysis and the slight gender imbalance does not alter our conclusion that erlotinib fails to prolong SOD1 mouse lifespan.

**Table 2:** Censoring information for survival study: animal n by cause of death/euthanasia for SOD1 Tg mice.

SOD1 Tg mice only				
Cause of death/euthanasia	Vehicle	Erlotinib	Total	Censored?
Loss of righting reflex (within 15s)	14	25	39	no
Loss of 20% body weight (from peak weight)	8	5	13	no
Both the above	5	6	11	no
Found dead (possible bad injection)*	15 (3F,12M)	7 (4F, 3M)	22	yes
Found dead (close to euthanasia)**	4	3	7	no
Euthanized due to injury (skin lesions)	0	1 (M)	1	yes
Lost >20% peak body weight but no other critical signs	0	1 (F)	1	yes
Total censored	**15** (3F,12M)	**9** (5F, 4M)	**24**	
Total uncensored	**31** (19F,12M)	**39** (19F,20M)	**70**	
**GRAND TOTAL**	**46** (22F,24M)	**48** (24F,24M)	**94**	

F: female; M: male.

* mouse was above critical weight and had normal righting reflex.

** mouse had already shown first signs of loss of righting reflex.

Although erlotinib did not extend survival, the treatment did improve motor function as assessed in multiple behavioral tests. Because disease progresses rapidly in the SOD1^G93A^ high copy line of mice, we scored neurological symptoms 3x/week so as to maximize the sampling, allowing us to detect even a small potential benefit. As part of the tri-weekly assessment mice were weighed, scored for tremor and hind limb extension, and tested on the wire hang.

**Figure 3:**
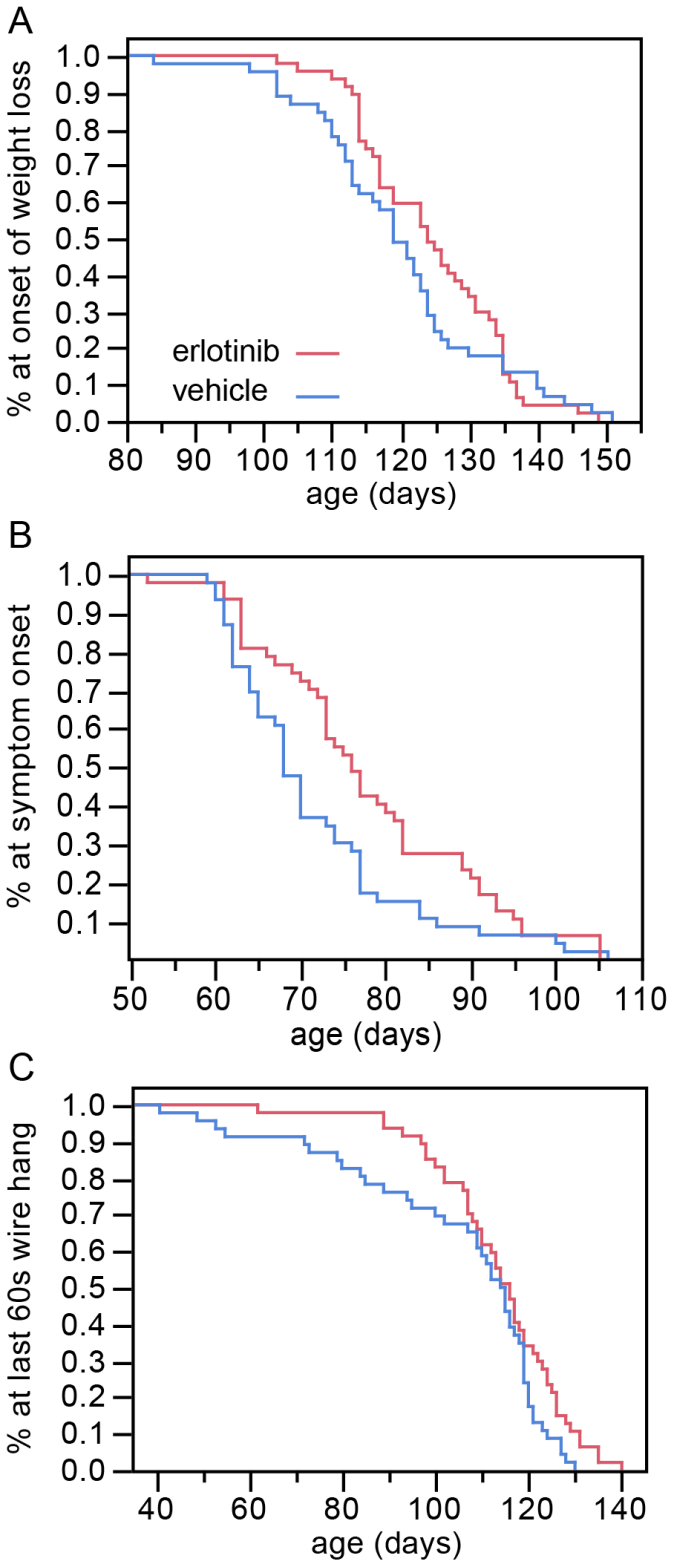
Erlotinib alleviates disease symptoms of SOD1 Tg mice. (A) Erlotinib trends toward delay in the age of onset of weight loss. This effect approaches significance, p = 0.0527 (Wilcoxon test). (B) Erlotinib delays the age of early symptom onset (p = 0.0061, Wilcoxon test). (C) Erlotinib improves the ability of mice to perform in the wire hang test, as shown by the age at which mice were last able to hang from the wire for 60s (p = 0.0479, log-rank test). The greatest difference between treatment groups is early on (∼40–110 days), suggesting a loss of efficacy of the drug as the disease becomes more severe.

Erlotinib trended towards delaying the onset of weight loss, although this did not reach significance (Figure 3A; vehicle mean = 120 days versus erlotinib mean = 125 days; refer to Table 3 for p values). The occurrence of early motor symptoms provides an even earlier measure of disease onset. We found that the loss of the hind limb extension reflex and/or the appearance of tremor (similar to ALS TDI neurological score of 1 [12]) were delayed by erlotinib treatment (vehicle mean = 72 days versus erlotinib mean = 78 days, Figure 3B; see Table 3 for p values). In the wire hang test, the erlotinib-treated group was able to perform the task for 60 full seconds at an older age than the vehicle group (vehicle mean = 104 days versus erlotinib mean = 114 days, Figure 3C; see Table 3 for p values). Interestingly, the beneficial effect for the wire hang was greatest at earlier time points, up until the convergence of the curves at day 110, suggesting that erlotinib loses efficacy as the disease progresses, at least for this motor ability. To provide some context to this time point, by day 110 the disease is readily apparent by casual observation, with SOD1 mice appearing thinner than wild-type counterparts, and displaying overt tremor and gait impairments.

**Table 3:** Time-to-event analysis of behavior and survival.

	Kaplan-Meier survival fit							
	Median time (days)			Mean time (days)			p value	
	V	E	Change	V	E	Change	Log-rank	Wilcoxon
**Early symptom onset**	68	76	8	71.7	78.1	6.4	0.0261	0.0061
**Onset of weight loss**	119	124	5	119.9	124.5	4.6	0.359	0.0527
**Last 60s wire hang**	115	116	1	104.4	113.7	9.3	0.0479	0.1196
**Survival (age at death)**	163	158	−5	160.8	159.4	−1.4	0.1725	0.4742

V: Vehicle; E: Erlotinib.

**Figure 4:**
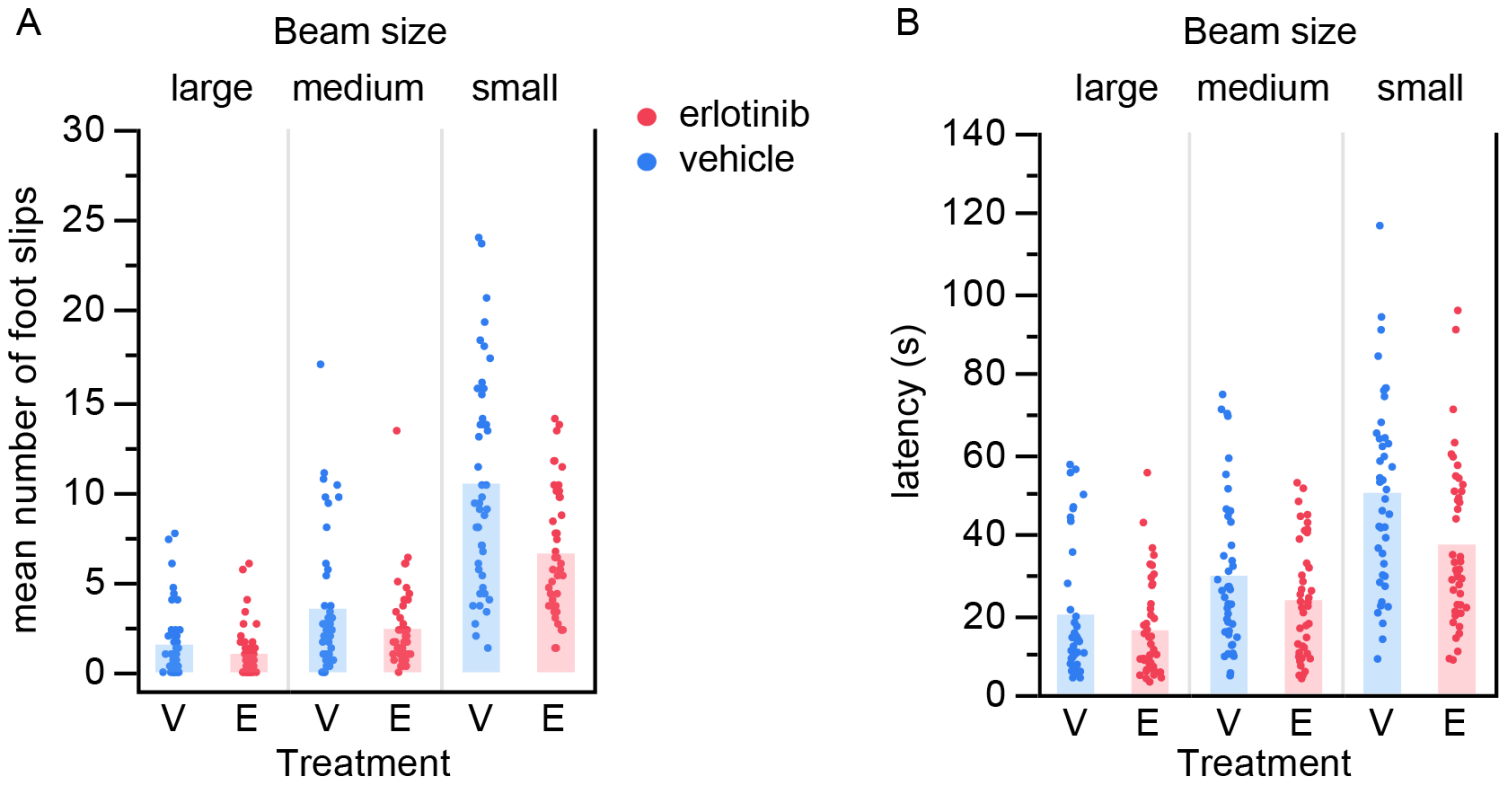
Erlotinib improves performance of SOD1 Tg mice on the balance beam. At ∼17 weeks of age, erlotinib-treated mice performed better on balance beams of 3 different sizes than vehicle-treated littermates, both for number of foot slips (A) and for latency to traverse the beam (B). Each point is the average of 3 trials per mouse. Bars represent group mean values. V – vehicle; E – erlotinib.

Finally, mice were tested on the balance beam at ∼17 weeks of age (actual age range: 118–125 days where mean = 120 days). We selected this time point because we had previously found that at this age the SOD1 mice are significantly impaired yet still able to perform the task. Erlotinib significantly improved the performance of SOD1 mice on the balance beam, both for number of foot slips as well as for latency to traverse the beam (foot slips p = 0.00023; latency p = 0.0097, likelihood ratio test for treatment effect, Figure 4A, 4B). The beneficial effect of erlotinib was observed on all three beam sizes tested. As a reference for the level of difficulty of this task for a healthy age matched littermate, examples of SOD WT data are shown in Figure S1.

### Erlotinib Reduces Phospho-EGFR Staining in Spinal Cord of SOD1 Tg Mice

To control for systemic presence of erlotinib in the dosed mice, we measured serum levels of erlotinib 6 and 24 hours post-injection (in the 3^rd^ and 4^th^ week of dosing of the histology study respectively; Figure S2A). Peripheral levels of erlotinib were in agreement with previous pharmacokinetic studies [23], including extremely low levels of erlotinib 24 hours post-injection, reflecting its short half-life.

We also measured erlotinib content in spinal cord of a separate group of mice (n = 15) which were dosed daily at 75 mg/kg IP for 8 days and harvested 2–3 hours post-last dose. The average erlotinib concentration in spinal cord was ∼800 ng/ml (Figure S2B). The ratio of erlotinib content in the CNS versus the periphery was 11%. We thus have evidence that our drug was crossing the blood brain barrier and reaching the spinal cord.

**Figure 5:**
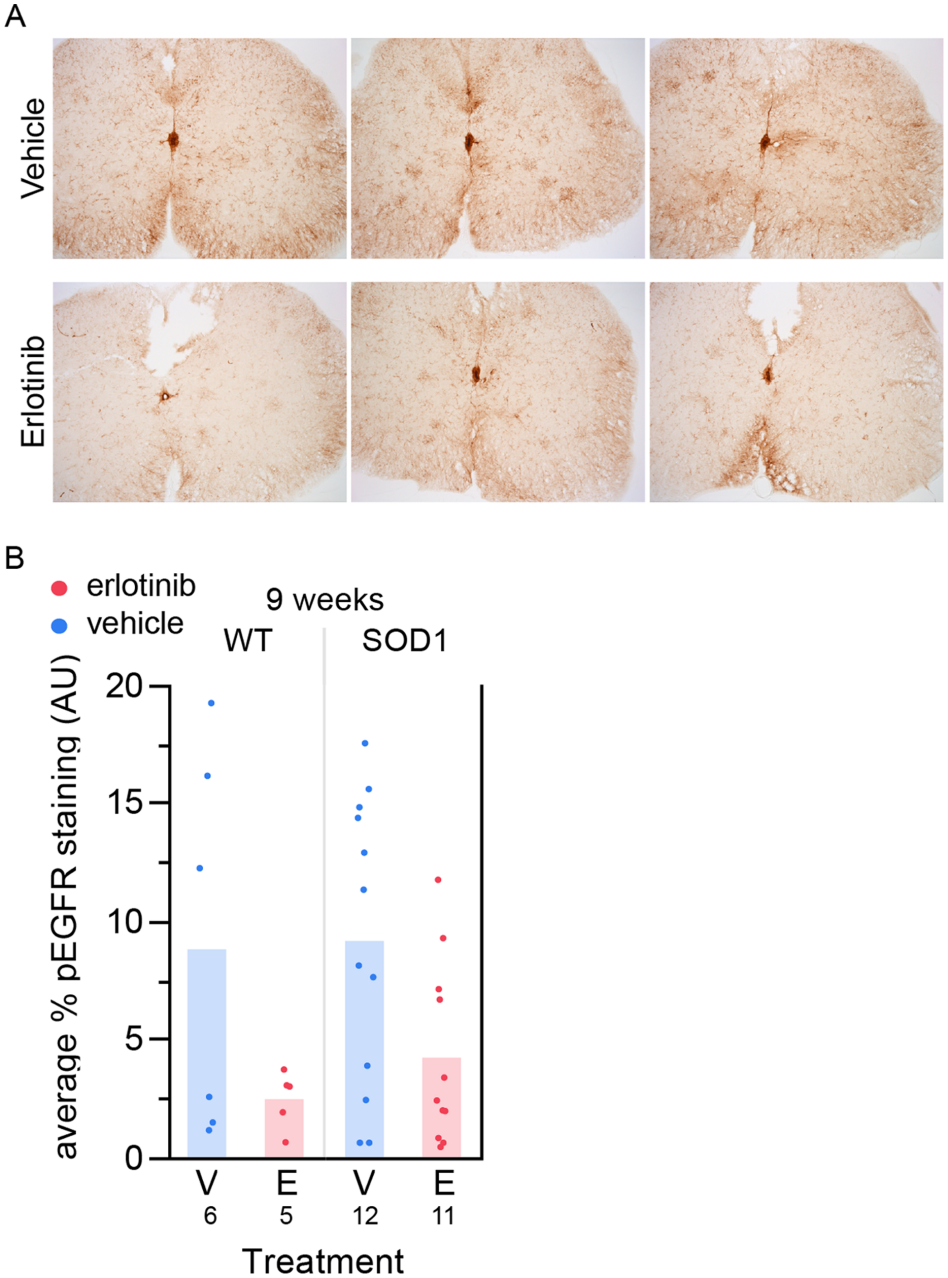
Erlotinib reduces anti-pEGFR staining in spinal cord. (A) Sample images from 6 different mice of anti-pEGFR staining in spinal cord. Top 3 images: vehicle-treated; bottom 3 images: erlotinib-treated. These images are not extreme examples from either group; they are representative of average levels of staining for each treatment group. (B) Quantification of staining using the Novus anti-pEGFR antibody in the 9-week lumbar spinal cord tissue, 24 hours post-last dose. Erlotinib significantly reduces the staining. p = 0.0124, one-tailed t test. The n/group is listed below each bar. V – vehicle; E – erlotinib.

To determine whether the drug levels observed in the CNS were biologically meaningful, we asked whether we could detect pharmacodynamic modulation of EGFR signaling in SOD1 mouse spinal cord. We immunohistochemically stained spinal cords from the histology study (24–26 hours post-last dose) with an anti-phosphoEGFR antibody (anti-pEGFR Tyr1068). The pEGFR staining appeared reduced in erlotinib- compared with vehicle-treated samples (Figure 5A). We quantified the amount of anti-pEGFR-positive staining in vehicle versus erlotinib-treated animals and found that erlotinib treatment significantly reduced the pEGFR staining (Figure 5B, 0.0124, one-tailed t test), suggesting that erlotinib was inhibiting EGFR signaling in spinal cord.

To confirm the specificity of the pEGFR antibody, we used it to stain EGFR KO tissue. We found that the antibody lacked specificity as demonstrated by positive staining in EGFR knockout mouse brain (Figure S3A). However, we were unable to directly compare adult spinal cord staining since EGFR KO mice are embryonic lethal [20]. These data suggest that, at least in the embryos, the antibody we were using was not perfectly selective for pEGFR. Indeed, this antibody had been generated using “a synthetic phospho-peptide corresponding to residues surrounding Tyr1068 of human EGFR” as immunogen (Novus NB110-56945 datasheet). An alignment of the region surrounding the equivalent tyrosine in mouse EGFR shows that the antibody is likely able to cross-react with EGFR family members ErbB2 and ErbB4 (Figure S3B).

Furthermore, potential off-target activity of erlotinib had previously been assessed across a panel of 287 distinct human kinases [24] [25]. Like many kinase inhibitors, erlotinib is not entirely specific to EGFR and can also bind ErbB2 and ErbB4 (with 74% and 68% efficiency respectively, versus 98% for EGFR). Due to the lack of specificity of the anti-pEGFR antibody, we cannot rule out that it is also detecting ErbB2 and ErbB4.

Finally, to confirm EGFR expression in adult mouse spinal cord we performed qRT-PCR on RNA from whole spinal cord of 15-week-old SOD1 Tg and WT littermate mice. We found that EGFR was indeed expressed in the spinal cord, as previously reported [15], although at reduced levels in transgenic mice versus wild type littermates (Figure S4).

Together, these data suggest that erlotinib was inhibiting EGFR in the spinal of SOD1 Tg mice, although we cannot rule out that it may additionally have been inhibiting ErbB2 and ErbB4.

### Erlotinib does not Protect Motor Neuron Synapses at the Neuromuscular Junction

To investigate whether the behavioral amelioration was the result of neuronal protection, we asked whether erlotinib treatment preserved motor neuron synapses at the neuromuscular junction (NMJ) of the gastrocnemius muscle.

Mice in this histology study (Figure 1B) were euthanized for tissue harvest 24 hours following the final dose at 9 weeks of age (n = 12 per treatment group) to assess protection of motor nerve endings at the NMJ. From our previous work in this mouse line, we know this age reveals significant denervation in SOD1^G93A^ transgenic versus wild type littermates.

**Figure 6:**
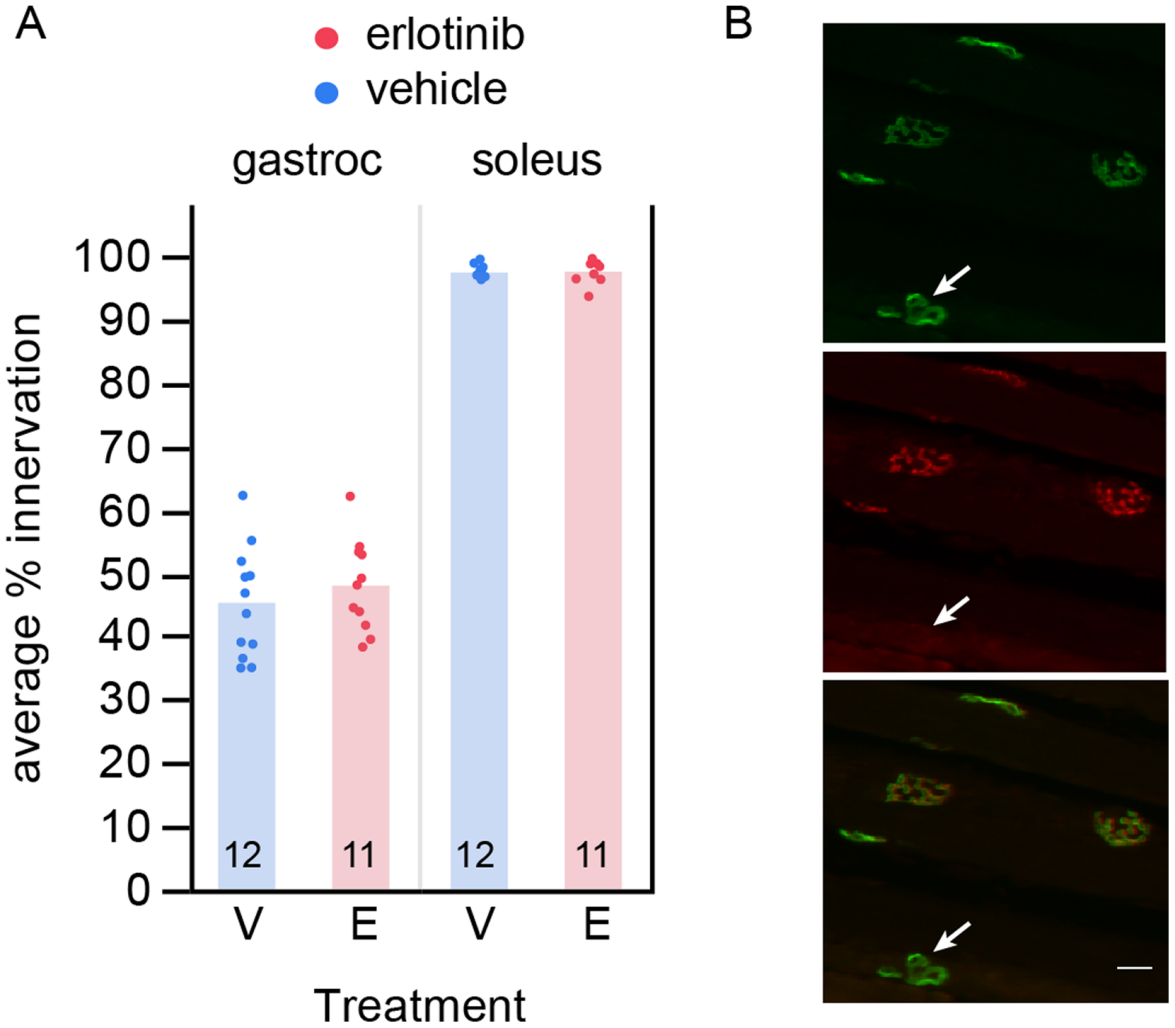
Erlotinib does not preserve motor neuron synapses in SOD1 Tg mice. (A) Erlotinib did not improve loss of motor neuron innervation to the gastrocnemius muscle, examined at 9 weeks of age. As a control for the innervation score, the soleus muscle, which is a slow-twitch muscle that is resistant to disease at this age, showed no loss of innervation. The n/group is listed below each bar. (B) Example image of innervated and denervated synapses. Alpha-bungarotoxin labels the postsynaptic site of each NMJ (green, top); VChAT labels the apposing presynaptic site of the NMJ (red, middle). Bottom: merge. Arrow points to the one denervated synapse among the synapses in this field (alpha-bungarotoxin-positive synapse lacking the presynaptic marker). Scalebar 20 µm. V – vehicle; E – erlotinib.

We found no difference between the erlotinib- and vehicle-treated groups for NMJ innervation. At 9 weeks of age (day 63) the average percent innervation at the NMJ in the gastrocnemius muscle of SOD1^G93A^ mice was 45.2% +/−2.3 (mean +/−SEM) for the vehicle group (n = 12) and 47.9% +/−2.4 for the erlotinib group (n = 12) (Figure 6A, left). As a control for our assay at the NMJ, we verified that the percent innervation in the soleus muscle was close to 100%, since this muscle has been shown to remain intact until later in disease progression, being made up of slow-twitch fibers that are more resistant to disease [4] (vehicle (n = 12): 97.4% +/−0.4; erlotinib (n = 12): 97.5+/−0.4; Figure 6A right). Figure 6B shows examples of innervated synapses and one denervated synapse.

Interestingly, despite the ∼46% synapse loss in the gastrocnemius of SOD1 Tg mice, motor neuron cell body counts were not statistically different from wild type at this age (Figure S5C). Motor neuron number was estimated by automated counting of cells positive for choline acetyltransferase (ChAT), the synthetic enzyme of acetylcholine (Figure S5A). The automated counts were verified against manual counts for a subset of slides. Both counts correlated well (R^2^ = 0.9, Figure S5B), thereby validating our automated method.

### Erlotinib does not Alter Astrocytic or Microglial Markers in Lumbar Spinal Cord

**Figure 7:**
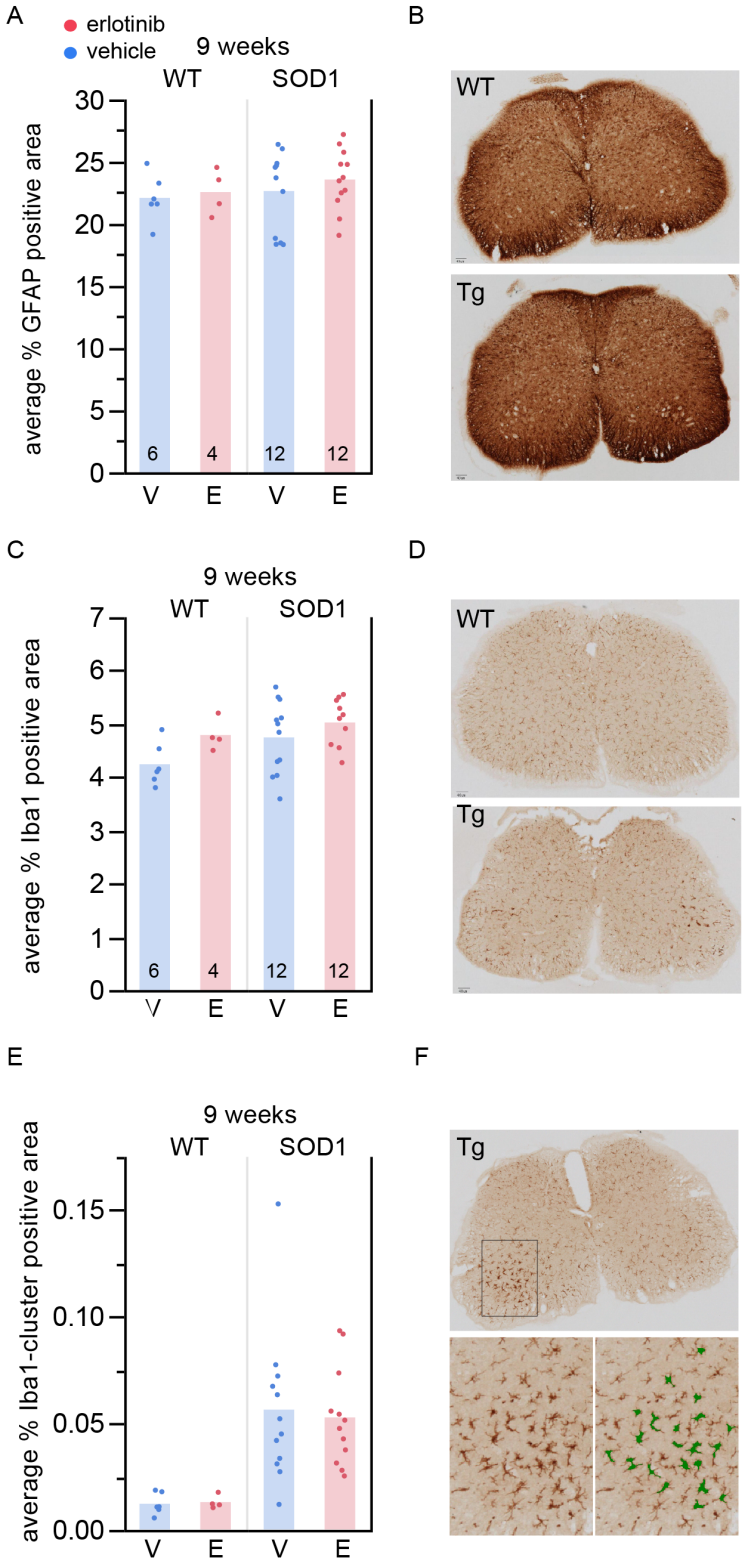
Erlotinib does not change the amount of astroglial or microglial staining in SOD1 Tg spinal cord. (A) Percent area stained positive for GFAP in 9-week spinal cord. The n/group is listed at the bottom of each bar. (B) Example images (top: WT; bottom: Tg). (C) Percent area stained positive for Iba1 in 9-week spinal cord. The n/group is listed at the bottom of each bar. (D) Example images (top: WT; bottom: Tg). (E) Percent area stained positive for Iba1+ enlarged cells or Iba1+ cell clusters in 9-week spinal cord. The n/group is the same as in (A) and (C). (F) Example images. Top: Tg; bottom left: closer view of microglia in the dorsal horn; bottom right: green mask overlay of region in bottom left showing the area detected by the automated algorithm as positive for Iba1+ enlarged cells or Iba1+ cell clusters. V – vehicle; E – erlotinib.

Since motor neuron disease in ALS involves a combination of autonomous and non cell-autonomous factors [26] we asked whether any glial changes could be detected upon erlotinib treatment as a possible explanation for the observed functional benefit. We quantified the amount of astrocytic and microglial staining at 9 weeks, after the mice had been on drug for 4 weeks, by measuring the amount of GFAP- or Iba1-positive staining in lumbar spinal cord (Figure 7). Erlotinib treatment had no effect on the amount of spinal cord astrocytes (Figure 7A,B) or microglia (Figure 7C–F).

Of note, at 9 weeks of age, although there was no difference between genotypes in total area stained positive for Iba1 (Figure 7C), there was a detectable genotype difference when the analysis included a minimum size cutoff in order to detect only enlarged Iba1+ cells or groups of cells in close proximity (Figure 7E; p = 0.0001, ANOVA). This analysis (Figure 7E,F) is thus a more sensitive measure capable of detecting even early microglial neuroinflammatory states.

## Discussion

In our survival study, while daily treatment with erlotinib from 5 weeks of age onwards failed to extend lifespan of SOD1 mice it did provide a modest yet significant symptomatic benefit as determined by multiple measures of disease onset and progression (loss of hindlimb extension and appearance of tremor, wire hang and balance beam tests). Although the amelioration of clinical signs was significant, it was modest and was insufficient to protect motor synapses or prolong SOD1 mouse lifespan. Thus, although erlotinib was reaching the CNS and having a pharmacodynamic effect in the spinal cord, we conclude that erlotinib is not efficacious in treating the SOD1 mouse model of ALS.

Although the behavioral benefits were modest, their observation at multiple time points in multiple measures increases our confidence that there was true symptomatic benefit and not a statistical anomaly. It is essential to test a large number of transgenic animals and monitor them frequently in order to detect small symptomatic changes with high confidence [17]. Since the histology and survival study dosing paradigms differed in their dose level by 15 mg/kg, we cannot exclude that we might have observed some motor synapse protection had the histology mice been dosed at 75 mg/kg as in the survival study, however this small difference in dose level is unlikely to explain the discrepancy between the behavioral observations and the lack of survival benefit. A more likely interpretation is that erlotinib treatment was sufficient to modestly ameliorate certain clinical signs but not to extend lifespan.

One limitation of these studies is that we were likely unable to inhibit EGFR signaling continuously throughout the course of disease. Our pharmacokinetic modeling suggests that for part of each day drug levels fell below the IC50 of 3 µg/ml defined for phospho-ERK (downstream effector of EGFR) in a xenograft cancer model [27]. However, the twice daily dosing that would have been required to sustain systemic drug levels would have been more stressful for this fragile mouse model. Indeed, the daily injections became challenging as the disease progressed, forcing us to discontinue dosing late in disease due to injection-related deaths. Nevertheless, the daily dosing regimen provided peak drug levels sufficient to inhibit EGFR activation as established in a xenograft model of cancer [27], and also allowed us to observe a pharmacodynamic effect in spinal cord in the present study (reduction in pEGFR staining). Furthermore, our chosen dose level was as high as possible based on the maximum tolerated dose of 50 mg/kg IV (intravenous) in mice and the recommended dosing level in humans (50 mg/kg PO (oral) in mouse is equivalent to 150 mg/kg in human, which is the dose prescribed for non small cell lung carcinoma).

It is possible that erlotinib provides symptomatic reprieve through a cell type other than the motor neurons, which is highly plausible in view of the evidence that the motor neuron degeneration in ALS is a combination of autonomous and non-cell autonomous factors [26]. For example, erlotinib could be acting through the glial cells that are abundant in the spinal cord of SOD1 Tg mice and thus modulating the neuroinflammatory response. Consistent with this idea, the pEGFR staining we observed was morphologically localized to glial populations and perhaps other cell types. However, we observed no treatment-related alteration in astroglial or microglial loads in spinal cord. This does not preclude there being a neuroimmune modulatory drug effect since it may not be measurable by glial-positive markers, but there were no major changes in glial load or activation.

In situ hybridization data in mouse spinal cord [28] suggests some low level of expression of EGFR in neurons and glia based on morphology and localization. The prior literature on the neuroprotective role of EGFR inhibition hypothesizes that the target cell type might be astrocytes [14] [15], but due to the likely lack of specificity of anti-phosphoEGFR antibodies used, the data remain inconclusive. More recent data on EGFR expression in astrocytes was available from published microarray datasets of FACS-sorted adult brain astrocytes [29] from two different in vivo models that induce reactive astrogliosis (middle cerebral artery occlusion (MCAO) and systemic lipopolysaccharide (LPS)-induced neuroinflammation). These data showed that EGFR is expressed at similar levels in astrocytes regardless of their activation state, suggesting it may not be a major player in the reactive astrocytic phenotype, contradicting the hypothesis in Liu et al., 2006 [14].

Also worth considering, EGFR is expressed in multiple epithelial tissues such as skin, liver, kidney, gastrointestinal tract and cornea. It is possible that by acting on a peripheral tissue the drug alleviates an aspect of SOD1 Tg mouse disease symptoms in the periphery that contributes to the overall health condition of the mouse, resulting in improved motor behavioral performance of erlotinib-treated mice compared with vehicle-treated littermates. This hypothesis is plausible since the mutant transgene is expressed throughout the body, not just in the CNS.

Finally, erlotinib, like most other small molecule inhibitors, has some off-target activity. It can inhibit other kinases including ErbB2, ErbB4, Flt3, Ret and Abl, with an efficiency greater than 60% [25]. Thus the behavioral amelioration could formally also be due to an off-target effect. For example, ErbB4 and Abl have both been implicated in neurodegenerative processes. Reportedly, systemic administration of the ErbB4 ligand neuregulin-1 protected dopaminergic neurons in a mouse model of Parkinson’s disease [30], and constitutively active neuronal c-Abl overexpression resulted in neurodegeneration [31].

In sum, we have presented evidence that erlotinib reached the CNS of the SOD1_G93A_ transgenic model and had a pharmacodynamic effect in spinal cord, yet its activity was insufficient to extend lifespan of ALS mice, despite producing small yet significant benefits in clinical signs. Although we cannot currently explain the modest amelioration in clinical signs, we conclude that erlotinib is not efficacious for the treatment of the ALS mouse due to the lack of lifespan extension and motor synapse protection. Given the lack of efficacy of erlotinib in this mouse model and the drug’s undesirable side effects, which include skin irritation and diarrhea, erlotinib does not appear to be a good clinical candidate for the treatment of ALS.

Our results suggest, however, that further elucidation of the pathways affected by daily erlotinib treatment could open up new avenues for slowing the course of this devastating disease.

## Supporting Information

Figure S1 **Balance beam data for SOD1 WT littermate mice.** As a reference, performance on the balance beam by healthy wild type littermate control mice: (A) number of foot slips, (B) latency to traverse the beam. Each point is the average of 3 trials per mouse. n = 3 per treatment group.
(TIF)

Figure S2 **Peripheral exposure of SOD1 mice to erlotinib.** (A) Serum concentration of erlotinib (ng/ml) in SOD1 WT and Tg mice bled at 6 or 24 hours post-dose in the histology study. The 6-hour time point occurred in the 3^rd^ week of dosing. The 24-hour time point occurred in the 4^th^ week of dosing. (B) Linear relationship between central (spinal cord) and peripheral (serum) exposure levels of erlotinib (ng/ml) in SOD1 mice for tissues collected 2–3 hours post-last dose.
(TIF)

Figure S3 **The anti-pEGFR antibody is not entirely selective for EGFR.** (A) The anti-pEGFR antibody detects a signal in E16.5 EGFR KO cortex. Green: anti-pEGFR; blue: DAPI (nucleic acid stain 4′,6-diamidino-2-phenylindole). Left: negative control for staining lacking primary antibody in EGFR KO tissue; middle: pEGFR staining in EGFR KO; right: pEGFR staining in EGFR WT littermate. (B) Peptide alignment of the region surrounding Tyr1068 (in bold) in EGFR, ErbB2 and ErbB4. Homologous amino acids are colored in black.
(TIF)

Figure S4 **EGFR mRNA is expressed in SOD1 Tg spinal cord.** qRT-PCR data showing -dC_T_ values for EGFR mRNA expression level in 15 week-old whole spinal cord homogenate of SOD1 WT vs Tg (n = 6 per genotype). EGFR expression is indeed detectable at the transcriptional level in mouse spinal cord. Further, these -dC_T_ values translate to a 0.56-fold expression level in Tg vs WT (p = 0.011, 2-tailed t test). Each point represents the average -dC_T_ for triplicate wells for RNA template from 1 animal. Boxplots: bars represent range between upper and lower quantiles; line represents the median; error bars represent the data spread.
(TIF)

Figure S5 **Motor neuron counts in 9-week old SOD1 Tg spinal cord are not different from WT.** (A) Example image of ChAT staining in Tg spinal cord, scalebar 100 µm. Lower left: higher magnification of ventral horn. Lower right: green dots show automated cell counts of region shown on lower left. (B) Linear fit between the manual and automated motor neuron counts (R^2^ = 0.9), showing the validity of the automated method. The automated counts consistently underestimate the number of observed neurons by about 25%, so the automated neuron counts should not be taken as absolute values. (C) Erlotinib did not preserve motor neuron cell bodies as stained by ChAT at 9 weeks. Each point represents the average motor neuron count per animal across 35 sections. Bars represent the mean. The n/group is listed at the bottom of each bar. V – vehicle; E – erlotinib.
(TIF)
