# Supplementary figures and images for: EGFR Inhibitor Erlotinib Delays Disease Progression but Does Not Extend Survival in the SOD1 Mouse Model of ALS
Source: PLoS One. 2013 Apr 26;8(4):e62342. doi: 10.1371/journal.pone.0062342 (PMC3637182; doi:10.1371/journal.pone.0062342)

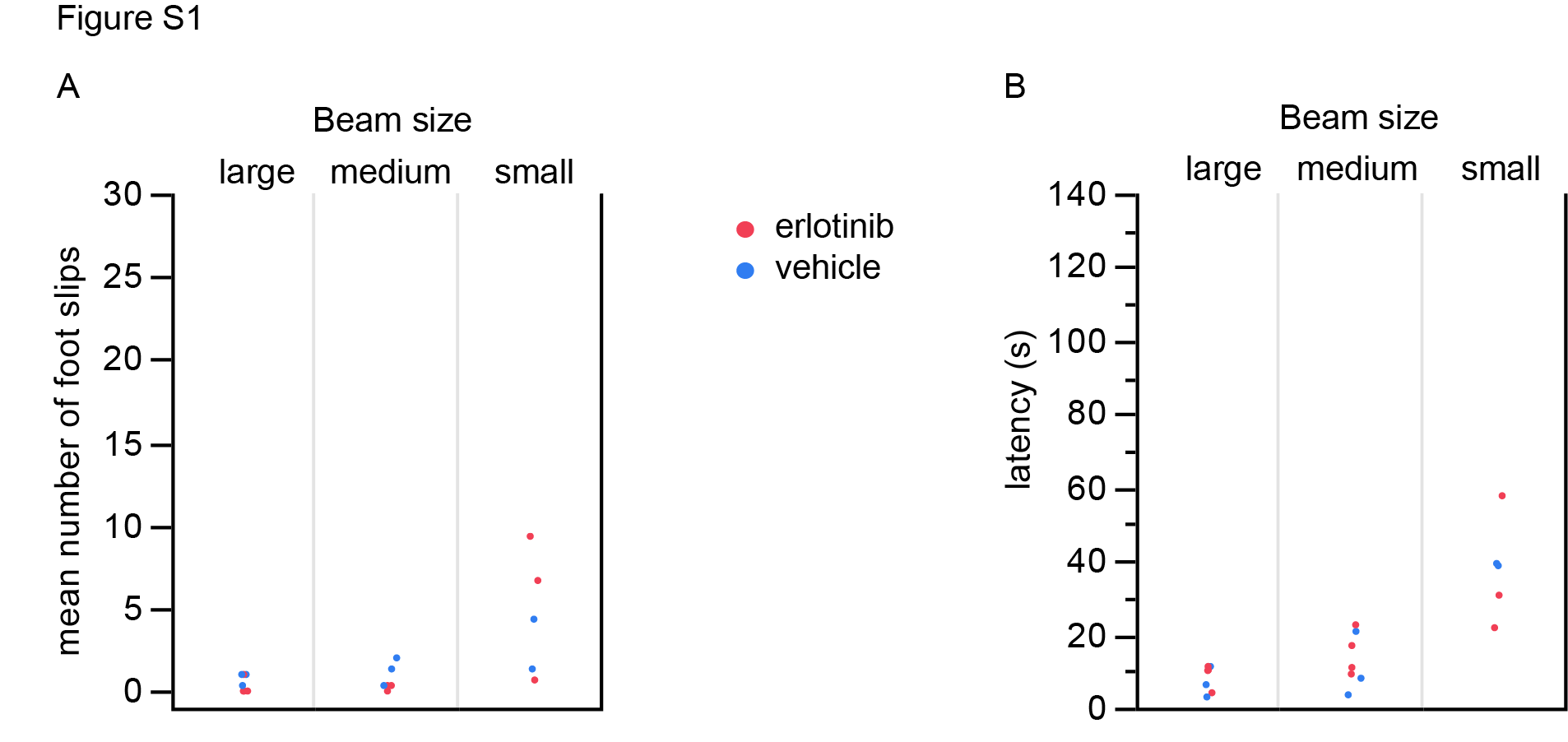

Supplement: Figure S1 — Balance beam data for SOD1 WT littermate mice. As a reference, performance on the balance beam by healthy wild type littermate control mice: (A) number of foot slips, (B) latency to traverse the beam. Each point is the average of 3 trials per mouse. n = 3 per treatment group. (TIF) [file pone.0062342.s001.tif]

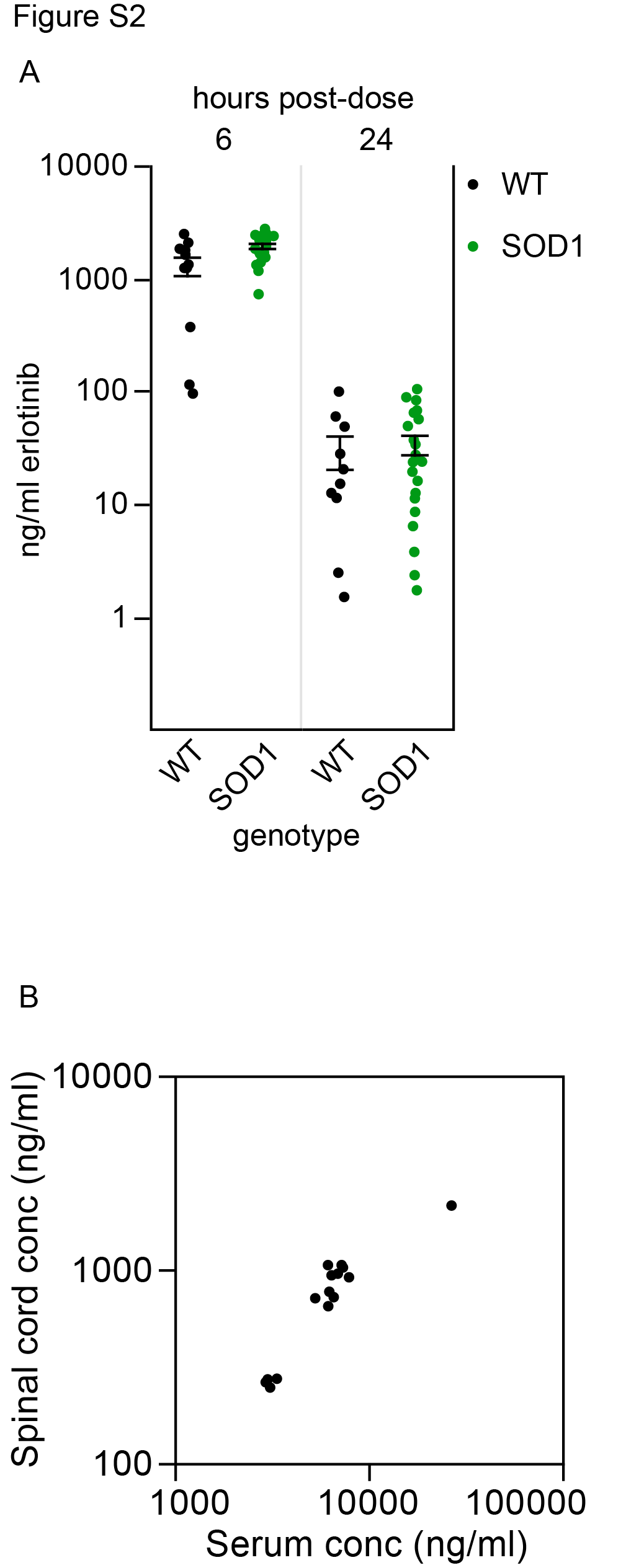

Supplement: Figure S2 — Peripheral exposure of SOD1 mice to erlotinib. (A) Serum concentration of erlotinib (ng/ml) in SOD1 WT and Tg mice bled at 6 or 24 hours post-dose in the histology study. The 6-hour time point occurred in the 3rd week of dosing. The 24-hour time point occurred in the 4th week of dosing. (B) Linear relationship between central (spinal cord) and peripheral (serum) exposure levels of erlotinib (ng/ml) in SOD1 mice for tissues collected 2–3 hours post-last dose. (TIF) [file pone.0062342.s002.tif]

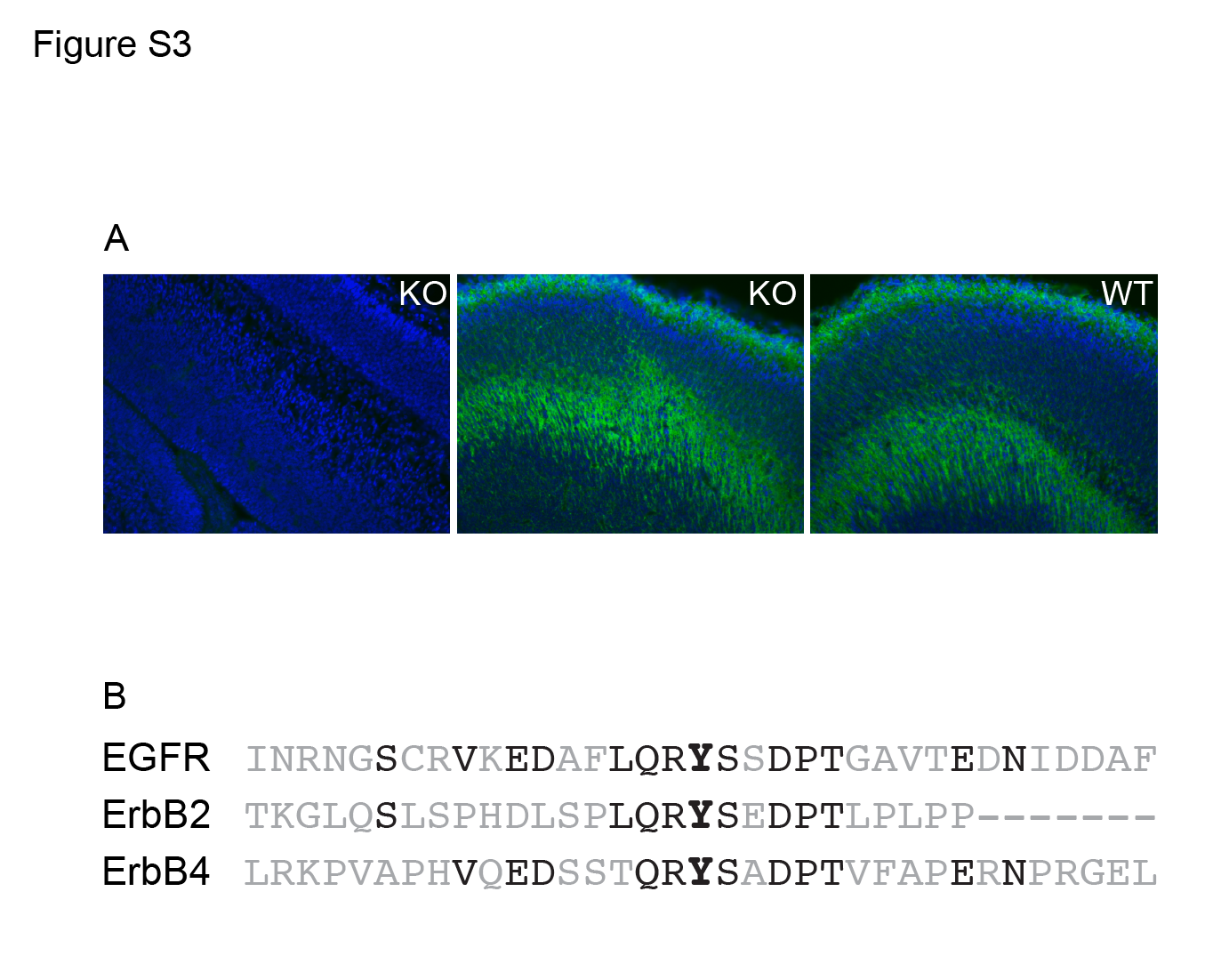

Supplement: Figure S3 — The anti-pEGFR antibody is not entirely selective for EGFR. (A) The anti-pEGFR antibody detects a signal in E16.5 EGFR KO cortex. Green: anti-pEGFR; blue: DAPI (nucleic acid stain 4′,6-diamidino-2-phenylindole). Left: negative control for staining lacking primary antibody in EGFR KO tissue; middle: pEGFR staining in EGFR KO; right: pEGFR staining in EGFR WT littermate. (B) Peptide alignment of the region surrounding Tyr1068 (in bold) in EGFR, ErbB2 and ErbB4. Homologous amino acids are colored in black. (TIF) [file pone.0062342.s003.tif]

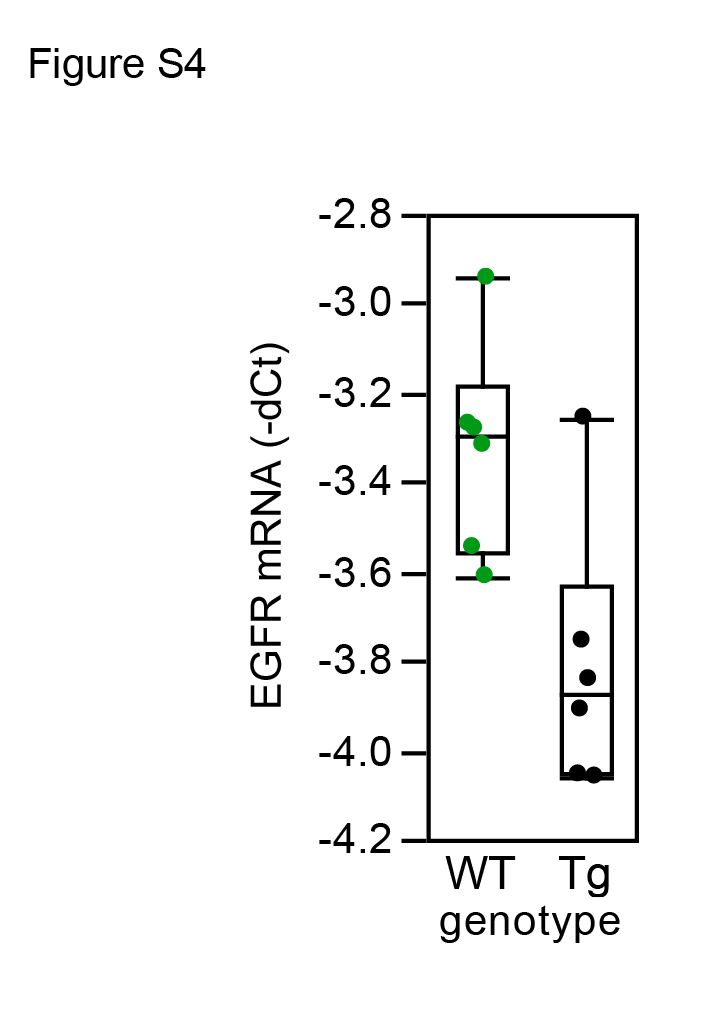

Supplement: Figure S4 — EGFR mRNA is expressed in SOD1 Tg spinal cord. qRT-PCR data showing -dCT values for EGFR mRNA expression level in 15 week-old whole spinal cord homogenate of SOD1 WT vs Tg (n = 6 per genotype). EGFR expression is indeed detectable at the transcriptional level in mouse spinal cord. Further, these -dCT values translate to a 0.56-fold expression level in Tg vs WT (p = 0.011, 2-tailed t test). Each point represents the average -dCT for triplicate wells for RNA template from 1 animal. Boxplots: bars represent range between upper and lower quantiles; line represents the median; error bars represent the data spread. (TIF) [file pone.0062342.s004.tif]

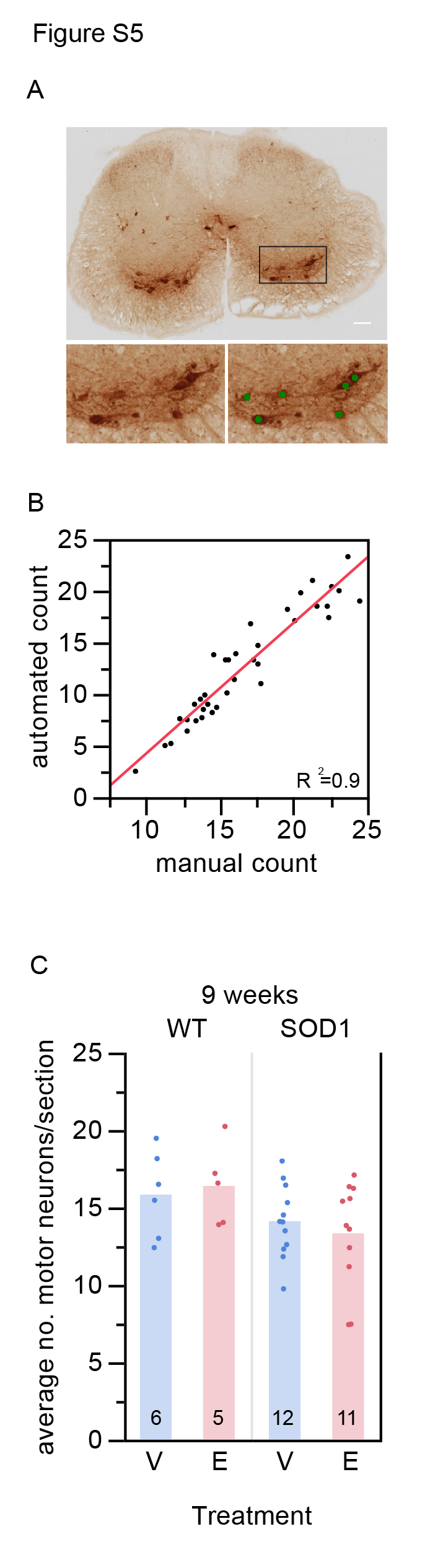

Supplement: Figure S5 — Motor neuron counts in 9-week old SOD1 Tg spinal cord are not different from WT. (A) Example image of ChAT staining in Tg spinal cord, scalebar 100 µm. Lower left: higher magnification of ventral horn. Lower right: green dots show automated cell counts of region shown on lower left. (B) Linear fit between the manual and automated motor neuron counts (R2 = 0.9), showing the validity of the automated method. The automated counts consistently underestimate the number of observed neurons by about 25%, so the automated neuron counts should not be taken as absolute values. (C) Erlotinib did not preserve motor neuron cell bodies as stained by ChAT at 9 weeks. Each point represents the average motor neuron count per animal across 35 sections. Bars represent the mean. The n/group is listed at the bottom of each bar. V – vehicle; E – erlotinib. (TIF) [file pone.0062342.s005.tif]
